# Keratinases: microbial sources, mechanisms, and industrial applications in waste valorization

**DOI:** 10.3389/fmicb.2026.1793191

**Published:** 2026-02-23

**Authors:** Yuqing Chu, Shan Chu, Fengna Hu, Mohan Huang, Haoran Lu, Zhiqiao Liu, Fenglian Shan, Xiaotang Chen

**Affiliations:** 1 College of Medical Engineering, Jining Medical University, Jining, China; 2 Affiliated Hospital of Jining Medical University, Jining, China

**Keywords:** biocatalysis, degradation mechanism, enzymatic properties, industrial applications, keratinase, protein engineering, waste valorization

## Abstract

Keratinous waste, a major by-product of agriculture and animal husbandry, is produced in massive quantities and is notoriously recalcitrant to degradation. With the expansion of the poultry and livestock industries, keratinous waste accumulation (e.g., feathers, hooves, and horns) has become a pressing environmental concern. Keratin’s highly cross-linked disulfide bond structure is resistant to breakdown by common proteases. Keratinase, a specialized protease capable of specifically degrading keratin, has emerges as a pivotal tool for the valorization of keratinous waste, demonstrating significant potential in waste management and resource recovery. This review systematically summarizes the enzymatic properties, mechanisms of action, and microbial sources of keratinases. It elaborates on innovative keratinase applications in waste valorization (including biogas production, the generation of bioactive peptides and amino acid feedstocks, and bioplastic manufacturing) and green industries (including leather and textile processing), as well as in the pharmaceutical, cosmetic, and detergent sectors. This review provides an in-depth discussion of the major challenges hindering industrial-scale keratinase application, including low heterologous expression efficiency and insufficient stability under industrial conditions. Finally, it outlines future research directions, encompassing protein engineering, artificial intelligence (AI)-assisted design, and multi-enzyme synergistic catalysis systems, aiming to offer forward-looking theoretical insights for advanced keratinase development and industrial application.

## 1 Introduction

Keratin is a structurally stable, disulfide-rich fibrous protein that is abundant in agricultural and livestock waste such as feathers, hair, and hooves. The rapid global development of the poultry and livestock industry has led to the annual generation of tens of millions of tons of keratinous waste materials. The recalcitrant nature of keratinous waste presents a pressing dual challenge: environmentally, its slow natural decomposition and conventional disposal methods (e.g., landfilling, incineration) lead to long-term pollution and resource wastage (Wen et al., 2020); economically, it represents a significant untapped reservoir of protein and amino acids, with high costs associated with its management and lost valorization potential. The rapid expansion of the poultry and livestock industries exacerbates this issue, making efficient waste-to-resource conversion an urgent priority. Keratinases are primarily secreted by microorganisms, including bacteria (Cedrola et al., 2012), actinomycetes (Jaouadi et al., 2010), and fungi (Kanoksilapatham et al., 2016). Within the protease superfamily, keratinases belong to the serine protease or metalloprotease families. Due to their unique mechanism of action, keratinases can efficiently cleave disulfide and peptide bonds in keratin under moderate temperature and potential of hydrogen (pH) conditions, enabling the rapid degradation and conversion of keratin. As such, keratinases have become a core research subject in the field of green biomanufacturing (Gupta and Ramnani, 2006).

In recent years, the application of metagenomics, structural biology, and synthetic biology has been accelerating advances in keratinase research. Since 2020, researchers have not only identified keratinases with unique properties produced in a wide range of extreme environments but have also made breakthroughs in understanding molecular degradation mechanisms, rational keratinase design, and the development of high-value-added products (Vidmar and Vodovnik, 2018). Numerous researchers have screened microbial strains that exhibit high keratin-degrading efficiency through enzymatic activity assays (Yang et al., 2022; Bokveld et al., 2023; Batool et al., 2020), aiming to promote the market-oriented application of keratinases and advance the sustainable development of green energy.

**Figure 1:**
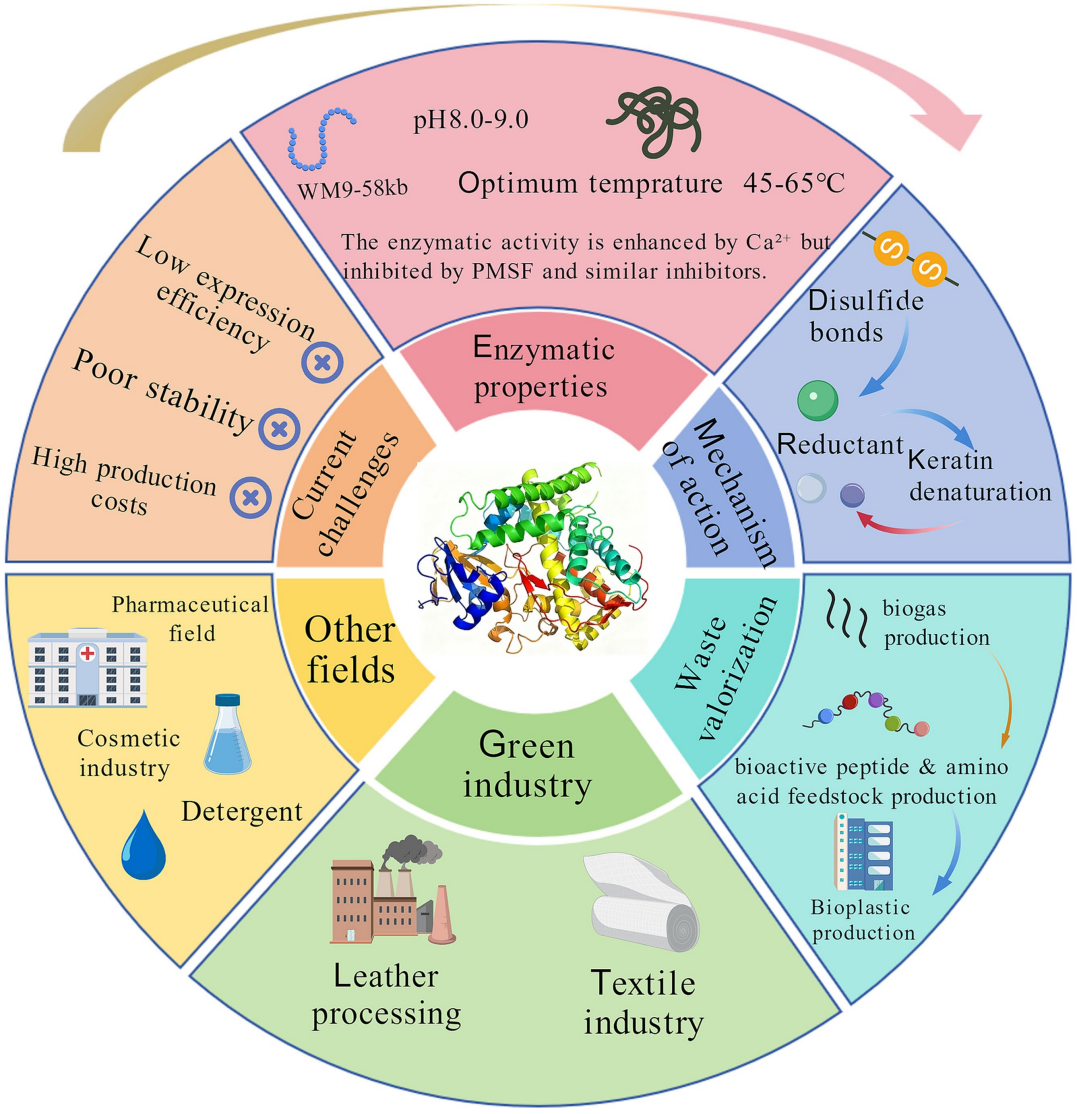
Graphical abstract.

This review aims to comprehensively assess the potential of keratinases in addressing these challenges. It systematically summarizes the enzymatic properties, mechanisms of action, and microbial sources of keratinases, as well as their innovative applications in waste valorization (such as biogas production, bioactive peptides and amino acid feedstocks, and bioplastic manufacturing), green industries (e.g., leather and textile processing), pharmaceuticals, cosmetics, and detergents. Moreover, this review further discusses major challenges hindering the industrial application of keratinases, including low heterologous expression efficiency and insufficient stability under industrial conditions (Figure 1). Finally, this review proposes future research directions involving protein engineering, AI-assisted design, and multi-enzyme synergistic catalytic systems, with the aim of providing a forward-looking theoretical reference for the in-depth development and industrial application of keratinases.

## 2 Literature search methodology

This review employed a targeted literature search strategy to identify and synthesize key research on keratinases, with a focus on their microbial sources, enzymatic properties, degradation mechanisms, and industrial applications. The search utilized key terms including keratinase, enzymatic properties, degradation mechanism, industrial applications, waste valorization, biocatalysis, and related terms. Searches were performed in three major English-language databases (Web of Science Core Collection, PubMed, and Scopus), and two leading Chinese databases (China National Knowledge Infrastructure, CNKI; Wanfang Data). Only literature published in English or Chinese was included, as these represent the predominant languages of academic publishing in this field; publications in other languages were excluded due to accessibility and translation constraints. The search covered the period from January 1997 to November 2025 to capture both foundational work and recent advances. Retrieved records were imported into EndNote reference management software, and duplicates were removed automatically based on title, author, year, and DOI, followed by manual verification. Literature selection was then based on the following criteria: direct relevance to keratinase properties, sources, mechanisms, or applications; publication in established, peer-reviewed journals; and robust experimental design with reliable data. Studies with incomplete data, findings not directly pertinent to the core themes, or redundant conclusions were excluded to maintain the quality and focus of the review.

## 3 Enzymatic properties of keratinases

### 3.1 General characteristics

In recent years, the extraction and characterization of keratinases have garnered increasing interest. Keratinases from different microbial sources exhibit distinct general properties, such as molecular weight, optimal pH, and temperature stability. The molecular weights of keratinases reported in recent studies vary widely, ranging from 58 kDa to as low as 9 kDa, although most recently reported keratinases are monomeric enzymes with molecular weights below 50 kDa. Most microbial keratinases exhibit optimal activity within the alkaline pH range of 8.0–10.0, indicating that they are predominantly alkaline proteases. However, some keratinases demonstrate stability and activity under acidic conditions or across a broad pH spectrum. For example, a novel keratinase expressed in *Escherichia coli* BL21(DE3) was shown to be stable within a pH range of 5.0–12.0 (Su et al., 2017). In terms of thermal characteristics, the majority of microbial keratinases display optimal activity between 45 °C and 65 °C. Notable exceptions exist, such as the thermostable keratinase GacK produced by *Geoglobus acetivorans*, which requires a temperature of 100 °C for optimal reaction efficiency and maximal keratin degradation. These variations in temperature adaptation are often attributed to the ecological niches and survival strategies of the source microorganisms. The key biochemical properties of selected microbial keratinases are summarized in Table 1.

**Table 1:** Biochemical characteristics of several microbial keratinases.

Keratinase	Source	Molecular weight (kD)	Optimum pH	Optimum temperature (°C)	References
Unnamed keratinase	*Bacillus zhangzhouensis*	42	9.5	60	Moridshahi et al. (2020)
Unnamed keratinase	*Chryseobacterium carnipullorum*	49.46	8.5	50	Mwanza et al. (2021)
kerA	*Bacillus licheniformis* PWD-1	30	8.0	45	Huang et al. (2017)
Extracellular keratinase	*Bacillus* LCB12	30.95	10.0	60	Tian et al. (2019)
Unnamed keratinase	*Bacillus aerius* NSMk2	9	8.0	45	Bhari et al. (2019)
Unnamed keratinase	*Escherichia coli* BL21 (DE3)	26	10.0	55	Su et al. (2017)
Unnamed keratinase	Agricultural waste	42.2	8.0	60	Nnolim and Nwodo (2020)
Exogenous keratinase	*Proteus vulgaris* EMB-14	49	9.0	60	Babalola et al. (2020)
GacK	*Geoglobus acetivorans*	58	10.0	100	Sittipol et al. (2021)
KER102 keratinase	*Bacillus*	30	10.0	50	Kshetri et al. (2020)
KERTYT protease	Feather-degrading strain *Thermo-actinomycetes* YT06	28	8.5	65	Wang et al. (2019)

### 3.2 Substrate specificity

#### 3.2.1 Substrate specificity of keratinases

Substrate specificity is a key enzymatic characteristic of microbial keratinases. These enzymes are capable of degrading both soluble substrates, such as casein, gelatin, and bovine serum albumin, as well as insoluble substances, including feathers, wool, and nails. Therefore, when characterizing a novel keratinase, it is essential to investigate its degradation efficiency and extent toward both soluble and insoluble substrates. Moridshahi et al. (2020) isolated a *Bacillus* strain from poultry feathers that produced a novel keratinase. Through optimization, purification, and identification, the enzyme was confirmed to be a serine protease with a molecular weight of 42 kDa. The researchers conducted experiments at a 1% (w/v) substrate concentration to evaluate the effect of different substrates on enzyme production and found that chicken feathers represented the optimal substrate for keratinase production by *Bacillus zhangzhouensis*, while cashmere wool and horse hooves were also identified as favorable substrates, demonstrating the ability of this microorganism to utilize both *α*-keratin and *β*-keratin substrates for keratinase synthesis. In another study, Qiu et al. (2022) investigated the substrate specificity of four previously uncharacterized Metalloprotease Family 36 (M36) proteases and observed that FoMep, PpMep, and AcMep generally displayed higher activity toward azocasein compared to other substrates, with FoMep showing the highest activity. However, when wool was employed as the substrate, AcMep performed best, whereas PpMep was most effective in degrading pig bristles or hooves. This variability in substrate preference among keratinases stems from differences in their binding-site architectures, including key amino acid residues, hydrophobic patches, and hydrogen-bonding networks that confer conformational selectivity for specific keratin types (de Kreij et al., 2000). The substrate-binding pocket directly recognizes and engages the three-dimensional structure of the substrate, providing spatial positioning for catalysis (Pei et al., 2024). Site-directed mutagenesis of these critical residues can alter substrate preference. Moreover, keratinase-producing microorganisms often inhabit keratin-rich environments, and long-term natural selection has shaped their enzymes to adapt to different keratin conformations.

#### 3.2.2 Structural basis of specificity: *α*-keratin vs. *β*-keratin

The distinct secondary structures and spatial conformations of α-keratin and β-keratin significantly influence the catalytic efficiency and substrate specificity of keratinases. *α*-Keratin (found in wool, hair, hooves, and nails) is dominated by α-helical coils that are tightly packed and highly cross-linked via disulfide bonds. In contrast, *β*-keratin (present in feathers and avian/scaly integuments) is characterized by β-sheet arrangements with looser packing and fewer disulfide linkages (Qiu et al., 2020). These structural differences directly affect enzyme accessibility and catalytic efficiency. The dense, disulfide-rich architecture of *α*-keratin creates considerable steric hindrance, often necessitating reductants or physical pretreatment to improve enzyme access and resulting in relatively lower hydrolysis rates. Conversely, the more open *β*-sheet structure of β-keratin presents less steric obstruction, facilitating enzyme binding and generally yielding higher basal hydrolysis efficiency. Consequently, enzymatic hydrolysis of α-keratin typically depends more heavily on reductive (e.g., β-mercaptoethanol, sulfite) or physical (e.g., thermal, ultrasonic) pretreatment to disrupt disulfide bonds and unfold the structure, whereas *β*-keratin is often more readily degraded with minimal pretreatment (Lene et al., 2016).

#### 3.2.3 Role of pretreatment in degradation efficiency

Beyond inherent enzyme specificity, keratin hydrolysis efficiency is strongly influenced by substrate pretreatment. For instance, high-temperature (121 °C) treatment or ultrasonication of feathers disrupts their native structure, leading to a two- to three-fold increase in keratinase hydrolysis efficiency. The addition of reducing agents (e.g., β-mercaptoethanol, sodium sulfite) cleaves disulfide bonds, further enhancing degradation (Pei et al., 2023; Kumari et al., 2024). The synergy between pretreatment (which improves substrate accessibility) and enzymatic action provides a crucial theoretical foundation for optimizing industrial keratin waste processing.

### 3.3 Specific activity

Specific activity serves as a key indicator for evaluating the activity, quality, and production process of keratinases. It is generally calculated as the space–time yield per unit catalyst surface area. Higher specific activity signifies more advanced keratinase production and purification processes, higher purity, and superior quality. With continuous research efforts and refinements in purification methodologies, the specific activity of microbial keratinases isolated over the past 5 years has shown varying degrees of improvement. Techniques such as ammonium sulfate precipitation, ultrafiltration, ion-exchange chromatography, gel filtration, and affinity chromatography, employed individually or in combination, have enabled the extraction of various novel keratinases with higher purity and better quality through more mature processes. This progress lays a solid foundation for subsequent industrial applications. The specific activities of several microbial keratinases are detailed in Table 2.

**Table 2:** Specific activity of several microbial keratinases.

Microbial keratinase	Specific activity (U/mg)	References
rKERDZ	66,600	Qiu et al. (2022)
Reconstituted bacmid with Ker-his-Flag	635	Mwanza et al. (2021)
Extracellular keratinase gene of *Bacillus* LCB12	9,813.2	Huang et al. (2017)
Keratinase from *Bacillus aerius* NSMk2	183.68	Tian et al. (2019)
Keratinase produced by *Anoxybacillus* sp. PC2	480.4	Reis et al. (2020)
KERA-71	96,700	Kerouaz et al. (2021)

### 3.4 Enzyme activity

Enzyme activity refers to the capacity of an enzyme to catalyze a specific chemical reaction, measured by the conversion rate under optimal pH and temperature conditions. For keratinases, this reflects the enzyme’s ability to degrade keratin. Beyond pH and temperature, factors such as metal ions, inhibitors, and surfactants also significantly influence enzymatic activity. Comparative studies indicate that Ca^2+^ generally enhances keratinase activity, whereas Phenylmethylsulfonyl fluoride (PMSF), Ethylenediaminetetraacetic Acid (EDTA), and certain heavy metal ions act as typical inhibitors, exerting mild to strong suppressive effects. The response to these agents can also serve as a diagnostic tool for enzyme classification. For example, the strong inhibition of a keratinase from a newly isolated bacterium—*Chromobacterium intermedium*—by both PMSF and EDTA indicates that it belongs to the metallo-serine protease family (Huang et al., 2017). The enhancers and inhibitors of various microbial keratinases are summarized in Table 3.

**Table 3:** Enhancers and inhibitors of several microbial keratinases.

Microbial keratinase (or source)	Enzyme class	Enhancement effect	Inhibition effect	References
*Bacillus zhangzhouensis*	Serine protease	Ca^2+^, K^+^, Na^+^, Mn^2+^	–	Moridshahi et al. (2020)
*Chryseobacterium carnipullorum*	Serine protease	Mg^2+^, Ca^2+^	PMSF	Mwanza et al. (2021)
LCB12	Metallo-serine protease	—	PMSF, EDTA	Huang et al. (2017)
*Bacillus aerius* NSMk2	Serine protease	K^+^, Na^+^, Ca^2+^, Mn^2+^, sodium sulfite, β-mercaptoethanol, dithiothreitol, ethanol, isopropanol, Tween-20, Tween-80	Hg^2+^, Ba^2+^	Tian et al. (2019)
KERA-71, KERB-19	Serine protease	Ca^2+^	PMSF, DFP	Kerouaz et al. (2021)
*Ochrobactrum intermedium*	Metallo-serine protease	Ca^2+^	PMSF, EDTA	Sharma and Kango (2021)
KBALT	Serine protease	Ca^2+^, Zn^2+^, Ba2^+^, Fe^3+^, Mn^2+^, Mg^2+^	Pb^2+^, Ag^2+^, Hg^2+^, PMSF	Pawar et al. (2018)

### 3.5 Stability

The stability of keratinases is influenced by multiple factors, including temperature, pH, metal ions, chemical additives, and protein engineering modifications. Numerous studies have reported the enhancement of keratinase stability using semi-rational or rational design approaches. Keratinase engineering efforts primarily target three key regions: the pro-peptide and C-terminal extension regions, which modulate overall enzyme conformation to optimize activity and stability, and the loop regions of the mature enzyme, where the introduction of molecular interactions such as hydrogen bonds or ionic bridges can increase structural rigidity. Additionally, modifications to substrate-binding sites, such as the mutation of specific amino acid residues within binding pockets, can directly influence enzymatic activity (Wang et al., 2023).

Furthermore, keratin exhibits strong thermal stability and can withstand feed pelleting temperatures. Elevated temperatures may also increase the keratinase enzymatic reaction rate. Studies have employed directed evolution to improve the thermostability of these enzymes. For example, Fu et al. (2021) utilized error-prone polymerase chain reaction (PCR) to conduct the directed evolution of a keratinase derived from *Bacillus licheniformis* CP-16. They successfully isolated mutant variants with enhanced thermal stability, thus broadening the applicability of the enzyme. Their analysis of the molecular basis for this improved stability provides new insights for the rational design of heat-resistant proteases. Alternatively, site-directed mutagenesis can be applied to increase molecular flexibility, thereby significantly enhancing catalytic activity at lower temperatures (e.g., 20 °C) while maintaining considerable thermostability.

## 4 Keratinase mechanisms and sources

### 4.1 Keratinase mechanisms

**Figure 2:**
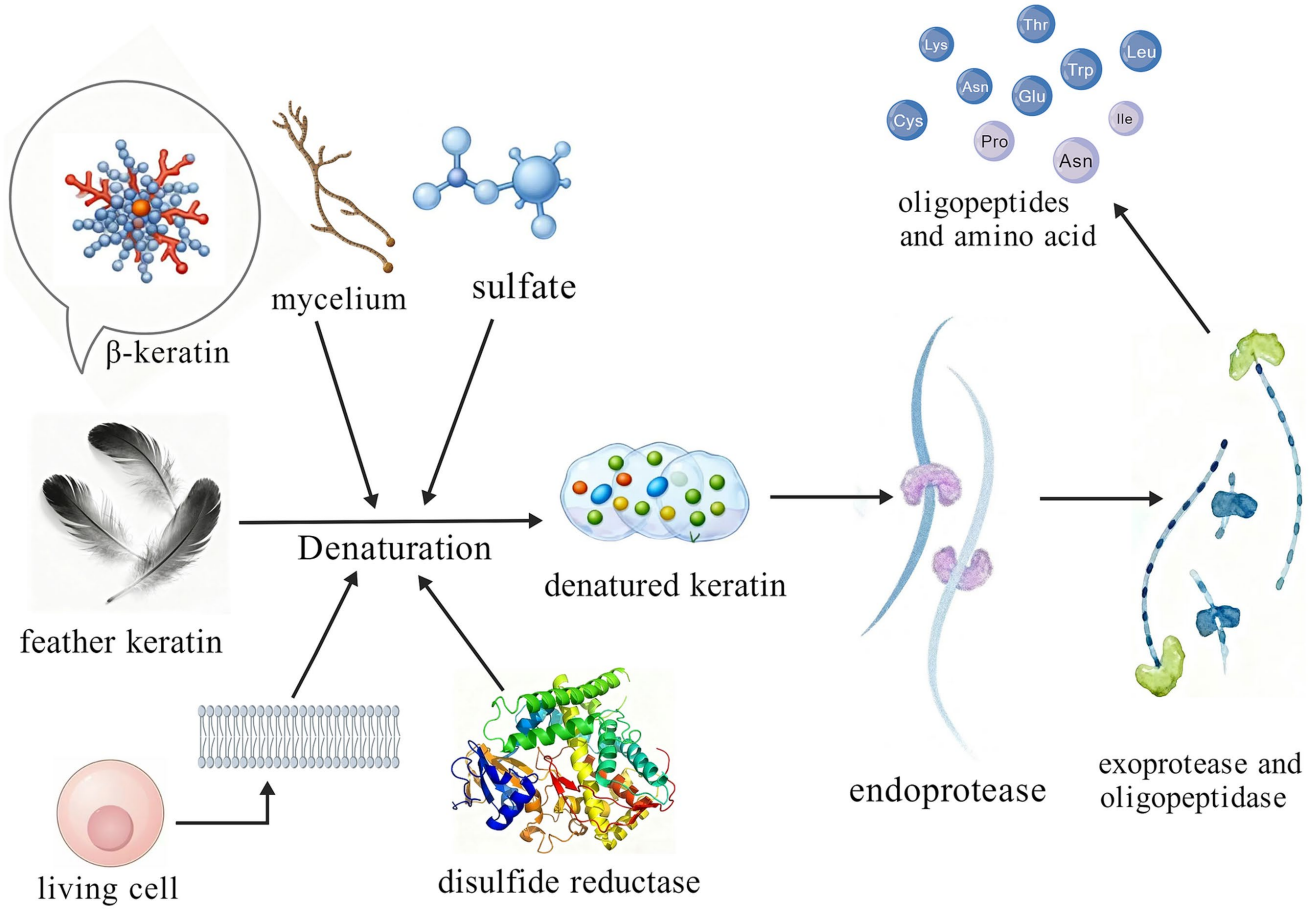
Keratinase disulfide bond reduction–proteolysis mechanism.

Industrial processes generate increasing amounts of keratinous waste composed of insoluble proteins, including feathers and hair. Due to their robust structure, these materials resist degradation by conventional proteases. The extensive disulfide bonds and cross-linkages within keratin form a highly rigid, cross-linked polypeptide framework, necessitating specialized biological recycling approaches. The two-step disulfide bond reduction–proteolysis mechanism represents a widely accepted model for keratin degradation (Figure 2) (Wang et al., 2023; Peng et al., 2019). According to this model, the initial step involves the cleavage of stable disulfide bonds (–S–S–) in keratin by reductive factors such as microbial disulfide reductases, reducing these disulfide bonds to free sulfhydryl groups (–SH). This critical reaction disrupts the chemical forces maintaining keratin’s rigid structure, converting it from an ordered crystalline state into a loose, denatured conformation and exposing previously hidden peptide bonds. In the subsequent step, a suite of keratinolytic enzymes, including endoproteases, exoproteases, and oligopeptidases, hydrolyze the exposed peptide bonds, ultimately breaking down the denatured keratin into absorbable oligopeptides and free amino acids. While the disulfide bond reduction–proteolysis model outlines the core degradation pathway, recent studies have proposed several cutting-edge theories emphasizing different aspects of the initial denaturation mechanism, particularly regarding how keratinase systems efficiently initiate the disruption of the native keratin structure.

#### 4.1.1 Theory physical pressure (hyphal penetration) theory

**Figure 3:**
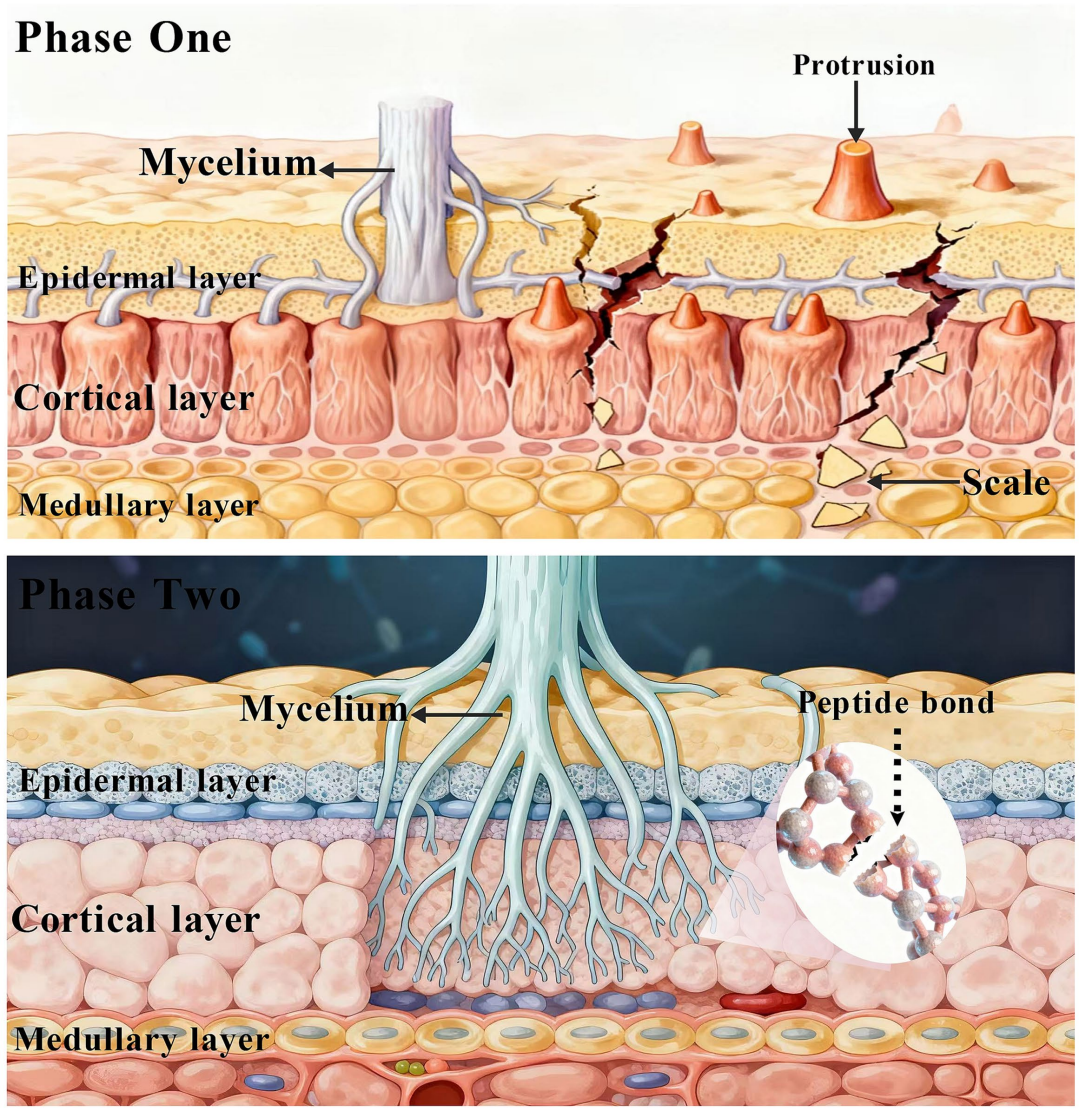
Mechanism of physical pressure theory.

The physical pressure theory primarily applies to filamentous fungi and certain actinomycetes, proposing that keratin’s structural integrity is compromised through the combined action of hyphal growth tension and mechanical penetration (Peng et al., 2021). As illustrated in Figure 3, the process unfolds in two key stages. Initially, mycelia attach to and grow along keratin fibers, generating tension that swells and ruptures the cuticle layer, causing surface exfoliation. Microscopic evidence (e.g., observations of hyphal wrapping around keratin fragments) suggests that this physical attachment may facilitate subsequent biochemical steps, such as disulfide bond reduction (Kaewsalud et al., 2023; Wang et al., 2015). Following surface disruption, mycelia penetrate into deeper cortical regions, where mechanical pressure exposes hidden peptide bonds for protease degradation. Notably, this physical mechanism operates in synergy with biochemical reduction, forming an integrated degradation strategy.

#### 4.1.2 Biofilm electrochemical theory

The biofilm electrochemical theory holds that intact, living cells and the presence of cell membranes are required to generate the necessary reductive capacity to cleave keratin disulfide bonds (Bockle and Muller, 1997). However, the underlying molecular mechanisms remain unclear. Studies have confirmed that washed *Streptomyces pactum* cells display significant disulfide bond reduction activity, whereas their culture filtrates lack this function (Bockle and Muller, 1997). Furthermore, keratinase enzymes or intracellular reducing substances alone are insufficient for complete feather degradation, and the presence of viable cells is indispensable (Ramnani et al., 2005).

#### 4.1.3 Thiolysis theory

**Figure 4:**
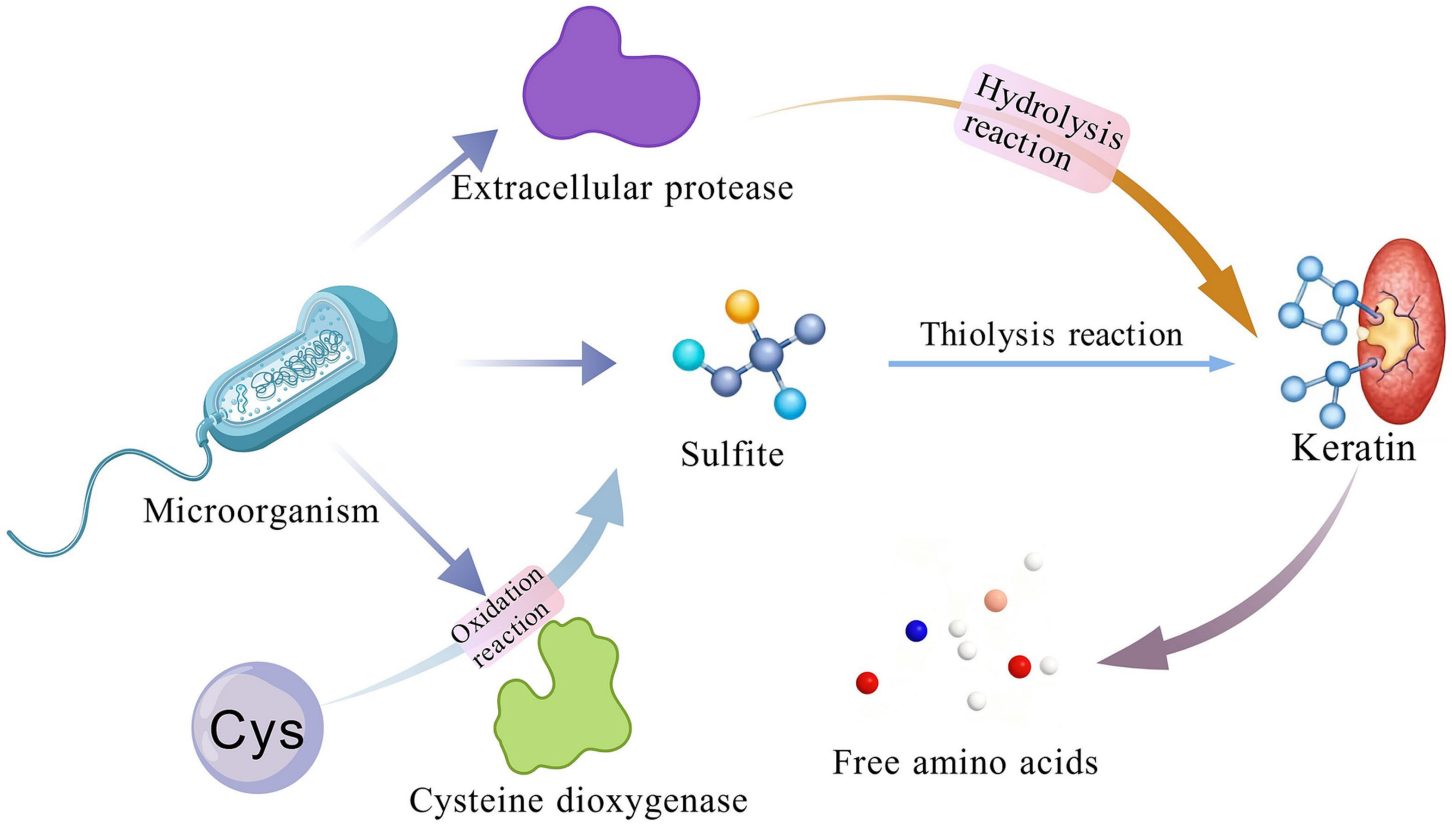
Mechanism of thiolysis theory.

The thiolysis theory represents a key mechanism for microbial keratin degradation. Its central premise involves the microbial secretion of thiol-based cofactors (such as *β*-mercaptoethanol and thioglycolate) or reducing agents such as sulfite, which cleave the disulfide bonds (S–S linkages) within keratin molecules. This disruption destabilizes the protein’s native three-dimensional structure, leading to keratin denaturation and consequently rendering the keratin more susceptible to hydrolysis by extracellular proteases (Grumbt et al., 2013) (Figure 4).

#### 4.1.4 Multi-enzyme synergistic system theory

The multi-enzyme system theory posits that efficient keratin degradation requires synergistic actions between keratinolytic proteases and accessory enzymes, such as disulfide bond reductases. A foundational study by Yamamura et al. (2002) demonstrated this principle using *Stenotrophomonas* sp. D-1, which co-secretes a serine keratinase and a disulfide bond reductase. *In vitro*, the combined use of these enzymes increased keratin hydrolysis rates by 50-fold compared to the protease alone. Notably, the reductase also enhanced the activity of other proteases (e.g., from *Bacillus subtilis*, trypsin, and proteinase K), underscoring its broad auxiliary role. Extending this concept, recent research by Kaewsalud et al. (2023) revealed a sophisticated synergistic mechanism between two distinct extracellular keratinases in *Thermoactinomyces vulgaris* TK1-21, offering novel molecular-level insights. This strain secretes both a 28 kDa serine protease (keratinase-I) and a 36 kDa metalloprotease (keratinase-II), which operate cooperatively in an “endo-exo” manner. Kinetic analyses show complementary substrate specificities: the serine protease exhibits high affinity for both *α*-keratin (Km = 0.69 mg/mL) and *β*-keratin (Km = 0.45 mg/mL), whereas the metalloprotease displays a preference for β-keratin (Km = 1.29 mg/mL) over α-keratin (Km = 1.40 mg/mL). This functional division of labor enables TK1-21 to efficiently degrade diverse keratinous wastes, achieving hydrolysis yields of 60–70%. This performance markedly surpasses that of single-enzyme systems, such as the keratinase from *Bacillus halodurans* SW-X, which exhibits relatively low activity (1.21–1.79 U/mL) against α-keratin.

#### 4.1.5 Keratinase catabolism via hydrophobic interactions in the urea cycle

Kang et al. (2021) investigated key metabolic pathways involved in keratinase activity, including amino acid metabolism, disulfide reduction, and the urea cycle through extracting enriched genomic DNA from *Chryseobacterium*, *Stenotrophomonas*, and *Pseudomonas* species and employing these three microbial consortia to reconstruct a composite genome. Keratin is composed of amino acids, which can be converted through a series of reactions into intermediates of the citric acid cycle, such as α-ketoglutarate or succinyl-CoA, for further amino acid metabolism. Upon genomic reconstruction and the annotation of sulfide and glutathione metabolic pathways, enzymatic screening revealed the presence of sulfate adenylyltransferase, adenylylsulfate kinase, and adenylylsulfate reductase—enzymes indicative of a complete dissimilatory sulfate reduction pathway. This suggested the presence of a fully functional disulfide reduction metabolism. Previous studies have established that urea denatures proteins via hydrophobic interactions, a process that necessitates four essential enzymes: argininosuccinate synthase, argininosuccinate lyase, arginase, and ornithine carbamoyltransferase. All four enzymes were identified in the reconstructed urea cycle established in this study, demonstrating that the microbial consortium could endogenously generate urea and leverage urea cycle-mediated hydrophobic interactions to support the catabolism of keratinases.

#### 4.1.6 Sulfite-initiated keratinase catalytic process

Keratinases play a crucial role in the decomposition of keratin; however, their activity declines significantly once separated from living cells. This observation suggests that viable cells may provide a cofactor that enhances keratinase efficiency. Peng et al. (2021) identified sulfite within living cells as the key agent initiating the keratinase catalytic process. Living cells regulate sulfite production through cysteine, thereby promoting keratin decomposition by keratinases and leading to keratin degradation. Following degradation, cysteine is released within the cells and converted into sulfite, forming a cyclic reaction that ensures thorough keratin breakdown. Further experiments revealed that coupling this cyclic catalytic system with keratinase-secreting cells enhanced the degradation capacity and efficiency of keratinases.

### 4.2 Keratinase sources

Keratinases are widely distributed in nature, with keratinase-producing microorganisms commonly isolated from keratinous waste such as feathers produced by poultry farms or waste from wool processing sites (Moridshahi et al., 2020). To date, more than 30 microbial species have been identified as keratinase producers (Gupta and Ramnani, 2006), primarily comprising fungi, actinomycetes (Bockle and Muller, 1997), and bacteria (Jaouadi et al., 2010), including *Bacillus* spp. (Moridshahi et al., 2020; Huang et al., 2017), *Lysobacter* spp. (Yan et al., 2024), and *E. coli* (Su et al., 2017). Furthermore, thermophilic microorganisms capable of secreting keratinases have been discovered in regions including India and Brazil, with these enzymes generally exhibiting higher optimal temperatures. Currently identified keratinases are predominantly classified as serine keratinases or metalloproteases, with serine-type enzymes being more prevalent. The M36 family is a protease family consisting of intracellular proteases. In previous research, Qiu et al. (2022) employed the Conserved Unique Peptide Pattern (CUPP) bioinformatics tool to classify this family into 11 groups. From Group 1, further analysis was performed on one known keratinase (from *Fusarium oxysporum*) and four previously uncharacterized proteases, PpMep, AcMep, PnMep, and NhMep, which originated from *Phaeosphaeria nodorum*, *Aspergillus clavatus*, *Pseudogymnoascus pannorum*, and *Nectria haematococca*, respectively, representing four distinct fungal lineages. The biochemical characterization of these proteases not only elucidated their catalytic properties but also provided new reference strains for future keratinase research.

The diverse mechanisms and microbial sources of keratinases provide a robust foundation for their practical deployment. Building upon this mechanistic understanding, the following sections explore how keratinases are harnessed for waste valorization and across various industries, transforming theoretical insights into tangible applications.

## 5 Keratinase-mediated waste valorization applications

### 5.1 Biogas production

**Figure 5:**
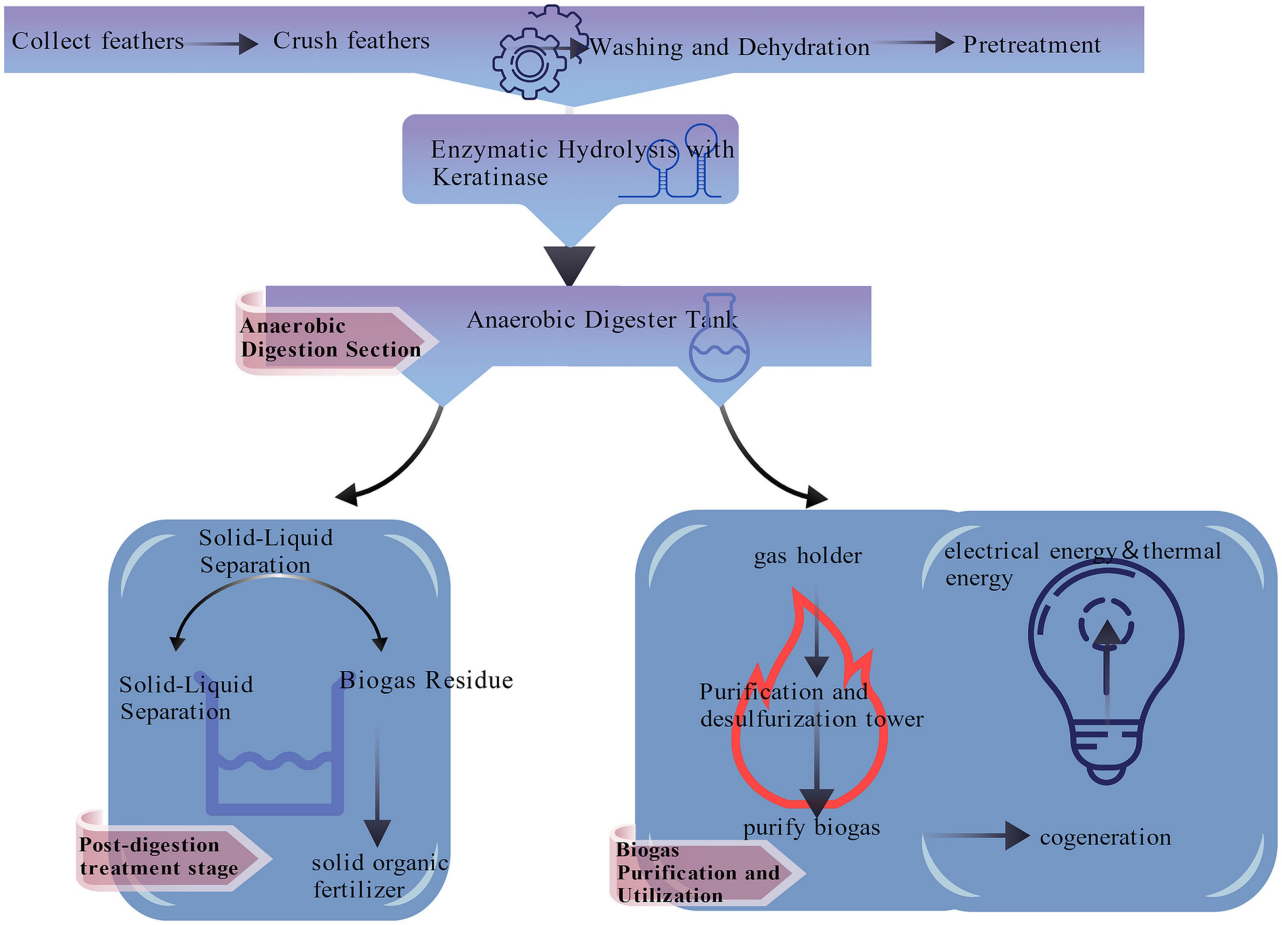
Bioconversion of keratinous waste (feathers) to biogas.

Global poultry production is projected to exceed 24.8 billion birds annually by 2030 (Qiu et al., 2020), leading to the immense accumulation of keratin-rich waste by-products such as feathers, beaks, and nails. Converting keratinous waste into biogas through keratinase-mediated hydrolysis represents an environmentally sustainable approach that promotes resource circularity. The transformation of feather waste into biogas comprises two key steps (Figure 5). In the first step, keratinase-producing strains hydrolyze keratin into oligopeptides and amino acids, which are subsequently converted into organic acids. In the second step, methanogenic archaea utilize these organic acids as substrates to generate methane via specific metabolic pathways. Throughout this process, keratinases play a critical role in accelerating keratin decomposition and enhancing the methane production rate. For example, Sun (2011) conducted a controlled experiment under thermophilic (55 °C) and anaerobic conditions to evaluate the impact of the keratinase-producing anaerobic strain M1-18 on methane yield from chicken feathers. By the fourth day of fermentation, the group inoculated with the keratinase-producing strain produced approximately 10 times more methane than the control, demonstrating the significant potential of keratinases in biogas production.

### 5.2 Production of bioactive peptides and amino acid feedstocks

Keratinases have shown significant application potential in the production of bioactive peptides and amino acid feedstocks. For example, Verma et al. (2017) demonstrated that the precise control of hydrolysis time, substrate concentration, and enzyme-to-substrate ratio can be employed to achieve the targeted production of specific functional peptides, such as antioxidant and antimicrobial peptides. Slaughterhouse cattle blood, a valuable protein resource, is often disposed of using methods that lead to resource waste and environmental pollution. To address this issue, Li et al. (2025) established an optimal process for efficiently converting cattle blood protein into functional protein ingredients using a keratinase (pH 9.0, 65 °C, enzyme dosage 3.75%, time 28 h). Under these conditions, a hydrolysis degree of 49.66% was achieved, yielding a high-quality hydrolysate rich in small-molecular-weight peptides (<500 Da, 45%). This product combines easy absorbability with strong antioxidant properties, confirming that it holds great potential as a high-value amino acid and bioactive peptide ingredient for applications in infant formula, health products, and antioxidant cosmetics. Furthermore, keratinases from *Bacillus* species also exhibit substantial potential in converting keratinous waste into high-value bioactive peptides and amino acid feedstocks (Gupta et al., 2025). A core advantage of these enzymes lies in their ability to efficiently hydrolyze the dense disulfide-bonded network in keratin, thereby degrading insoluble keratins—such as feathers and hair—into soluble peptides and free amino acids and consequently enabling the valorization of waste protein resources.

### 5.3 Bioplastic production

**Figure 6:**
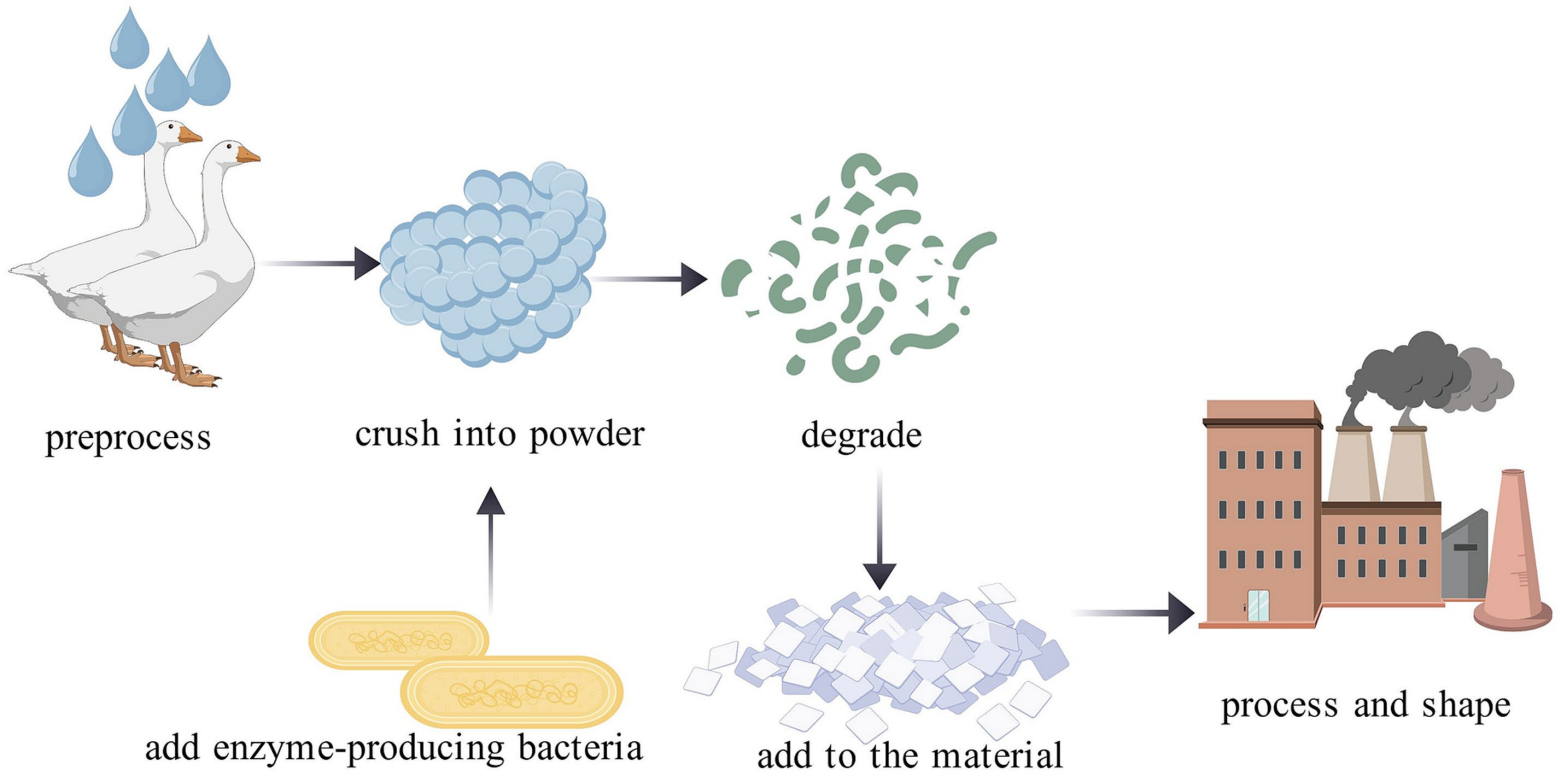
Keratinase-mediated bioplastic production process.

Replacing polluting, non-biodegradable petroleum-based plastics with non-toxic, biodegradable bio-based plastics represents a prevailing trend in the plastics manufacturing industry. Using chicken feathers as an example, the bioplastic production process is as follows (Figure 6). First, waste materials such as chicken feathers are thoroughly cleaned, followed by mild heating at 60 °C to remove residual lipids without damaging the keratin structure. The treated feathers are then ground into an 80–100-mesh powder. This powder is predominantly composed of insoluble keratin, which is characterized by a dense three-dimensional network stabilized by extensive disulfide bonds and hydrogen bonds that renders it incompatible with polymers. Keratinase-mediated biotransformation is therefore required. The feather powder is inoculated with keratinase-producing *Bacillus* strains (e.g., *Bacillus* sp. BAM3). The secreted keratinase is a serine protease that primarily hydrolyzes peptide bonds via its active-site serine residue and does not possess disulfide reductase activity (Kaewsalud et al., 2021). Cleavage of the disulfide bonds in keratin depends instead on intracellular reductive cofactors produced by the enzyme-producing strain. For instance, in *Bacillus halodurans* SW-X, sulfite generated through cysteine metabolism acts as the key reductant (Lene et al., 2016). This reflects a “single enzyme + intracellular cofactor” mode of action, rather than a bifunctional enzyme mechanism, and aligns with the microbial keratin degradation model proposed by Lange et al., wherein reductive factors and proteases collaborate sequentially.

After disulfide bond cleavage, the cross-linked keratin structure unravels and denatures, allowing the keratinase to hydrolyze the exposed peptide bonds. This process yields soluble low-molecular-weight peptides ranging from 10–20 kDa to ≥30 kDa (Kaewsalud et al., 2021). These keratin hydrolysates are then incorporated into a polymer matrix consisting of cassava starch (70% w/w, as matrix) and glycerol (30% w/w, as plasticizer), with glycerol also serving to adjust material flexibility. Similar to the strategy of Azevedo et al. (2019) for introducing functional components into starch-based materials, adding the hydrolysate at an optimal concentration of 0.10% (w/w) enables the formation of stable hydrogen-bonding cross-links within the starch–glycerol matrix. This enhances the tensile strength of the resulting bioplastic to 2.47 MPa (Kaewsalud et al., 2021). The mixture is subsequently processed—for example, by hot-pressing at 80 °C and 5–8 MPa for 30 min—to form a green and biodegradable bioplastic, designated B4 (Hassan et al., 2020). In a study by Alshehri et al. (2021), *Bacillus* sp. BAM3, a keratinase-producing strain, was utilized in chicken feather-based bioplastic production. Enzymatic pretreatment bioconverted feather waste into soluble keratin peptides, which were integrated into a bio-based polymer to form a keratin–polymer composite network. By controlling the extent of enzymatic hydrolysis, the material properties were optimized, yielding a keratin-based bioplastic that demonstrated satisfactory performance in tensile tests. This work provides a valuable reference for the market-oriented application of such bioplastics.

## 6 Green industry

### 6.1 Application in leather processing

The leather and textile industries require multiple steps—such as dehairing, deliming, bating, and degreasing—to convert raw hides (e.g., untreated pigskin or cattle hide with hair) into semi-finished products. These intermediates are further processed through sewing and cutting to produce everyday items such as shoes, hats, and belts. Although the tanning industry is recognized as an economic pillar in many countries, wastewater discharged from tanneries contains various recalcitrant pollutants, drawing increasing environmental concerns (Lofrano et al., 2013). The use of keratinases for dehairing presents a green and biodegradable alternative that is environmentally benign and does not damage the leather grain. In experiments conducted by Akhter et al. (2020), dehairing performed with keratinases was compared with conventional chemical treatment on identical substrates, revealing that keratinase-based dehairing was more thorough, without compromising the integrity of the leather layer. Recent studies have further validated the technical advantages and industrial potential of keratinases. For example, a research team from Jiangnan University developed a composite enzyme system combining keratinase with *α*-amylase and lipase for processing cattle hides. This system achieved a dehairing rate exceeding 95%, while reducing the total suspended solids (TSS) and chemical oxygen demand (COD) in wastewater by 91 and 43%, respectively, compared to the traditional lime-sulfide process. The resulting wet-blue leather met national quality standards (Niu et al., 2025). Similarly, the keratinase from *Bacillus subtilis* ES5 has been implemented successfully in Ethiopian tanneries. Operating at room temperature and pH 8.0, the crude enzyme preparation (activity: 317.34 U/mL) completely dehaired goat/sheep skins within 12 h and cattle hides within 16 h. Relative to the lime-sulfide method, the enzymatic process reduced key wastewater parameters substantially: biochemical oxygen demand (BOD) by 57.39%, COD by 70.53%, total dissolved solids (TDS) by 93.59%, TSS by 94.90%, and total pollution load by approximately 79.10% (Getachew et al., 2023). In another advance, a cold-adapted keratinase from *Penicillium oxalicum* AUMC 15084 enabled the dehairing of goat skins at a low temperature of 20 °C. This approach eliminated the need for sulfide while reducing energy consumption by approximately 60% (Abdel-Latif et al., 2025). Such progress underscores the potential of keratinases as a core technology for cleaner leather production.

### 6.2 Application in the textile industry

In the textile sector, treating wool with keratinases can significantly enhance its smoothness and softness without compromising the fiber’s intrinsic structure, thereby improving the comfort of wool-based textiles. The roughness often associated with keratin-containing fibers primarily stems from the scaly cuticle edges and residual impurities such as lanolin. Under moderate environmental conditions (temperature 35–50 °C, pH 7–9), keratinases induce the mild degradation of these surface asperities and residual contaminants while concurrently repairing micro-damage on the fiber surface, resulting in a finer fabric handfeel. When wool fabric was treated with keratinase immobilized on *β*-cyclodextrin, scanning electron microscopy (SEM) revealed that the enzyme specifically occupied the base of the cuticle scales. This prevented fiber entanglement, thereby improving the smoothness and stability of the fabric without damaging the wool fibers. This indicates that keratinases not only possess effective cleaning capabilities but also exert a protective effect on textile fibers, enhancing both softness and durability (Zhang et al., 2025). Furthermore, keratinases can act synergistically with other enzymes, such as transglutaminase, to improve the tensile strength of wool (Cortez et al., 2003), thus enabling its use in the production of more complex patterned textiles.

Keratinases have also contributed to technological advancements in the shrink-resistant finishing of wool. The scaly cuticle layer of wool is rich in disulfide bonds and presents a major barrier to dye uptake (Li et al., 2013). Moreover, this scaly surface structure is the primary cause of felting during washing. While the dichloroisocyanurate (DCCA) chlorination method can mitigate felting, it releases harmful chlorine-based gases and leaves residual formaldehyde, posing risks to human health. Treating wool with keratinase (20 U/mL, pH 9, 30 °C) for 12–24 h enables the targeted hydrolysis of the surface keratin scales, smoothing the fiber and enhancing its machine-washability (felting resistance), with significantly less strength loss compared to traditional DCCA chlorination (Park et al., 2023). Wastewater from this enzymatic process displays markedly reduced COD and BOD, aligning with the Oeko-Tex® Standard 100 for eco-textiles. Experimental results indicate that keratinase-treated wool fabrics can achieve a felting shrinkage rate of less than 5%, while retaining over 85% of fiber strength, thus successfully balancing environmental sustainability with fiber integrity.

## 7 Innovative keratinase applications in other fields

### 7.1 Potential applications in the pharmaceutical field

**Figure 7:**
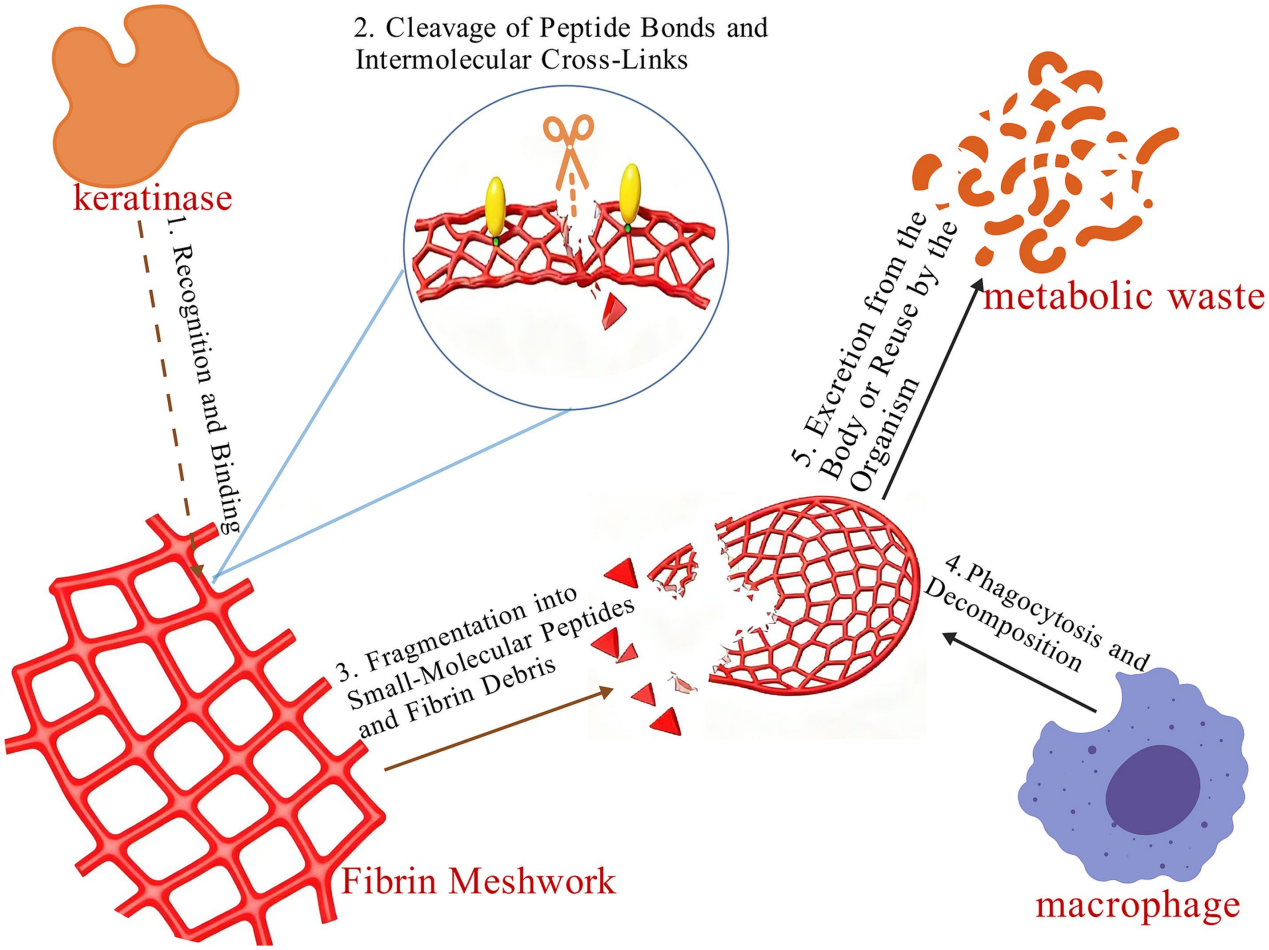
Process of thrombus dissolution by keratinase.

Keratinases show emerging potential in the treatment of skin diseases. For example, utilizing terbinafine gel in combination with a keratinase results in significantly higher therapeutic efficacy against tinea corporis compared to the use of terbinafine gel alone (Lu et al., 2018). Similarly, the combination of antifungal agents with keratinases has demonstrated notable effectiveness in treating onychomycosis (Rai et al., 2018). Calluses, commonly found on the palms and heels, are primarily composed of keratin, making keratinase-based debridement a common therapeutic approach. Due to their ability to hydrolyze prion proteins, keratinases have also drawn attention as potential agents for the treatment of prion disease (Spyros, 2003). Furthermore, keratinases play a significant role in nanoparticle synthesis (Ye et al., 2020). As a novel drug delivery system, nanoparticles are promising for their small size and ability to cross the blood–brain barrier. In wound management and tissue engineering, bioactive peptides and keratin nanoparticles derived from feather hydrolysis have shown potential in promoting wound healing and modulating immune responses (Zhang et al., 2023). Acne, a chronic inflammatory condition of the pilosebaceous units, commonly presents as comedones, papules, and pustules on the face during adolescence. This condition is characterized by excessive sebum production, which leads to pore blockage and subsequent inflammation. Leveraging their ability to degrade sebum, keratinases have been incorporated into topical acne treatments, such as in commercially available products including Keratoclean Sensitive PB and Keratopeel PB (Spyros, 2003). Additionally, enzymatic hydrolysates of chicken feathers have been utilized as a culture medium to produce bacterial cellulose with a fine network structure, offering a high-quality material for tissue engineering scaffolds and advanced wound dressings (Goda et al., 2022). Furthermore, a research team from Jiangnan University demonstrated that a recombinantly expressed keratinase could effectively degrade fibrinogen and dissolve blood clots. This enzyme cleaves peptide bonds in fibrin and likely disrupts intermolecular crosslinks, leading to the breakdown of the thrombus network (Liu et al., 2024) (Figure 7).

### 7.2 Applications in cosmetics

In the field of cosmetics, keratinases have emerged as key functional ingredients due to their exceptional substrate specificity, mild action, and biocompatibility. Keratinases excel as biological exfoliating agents in skincare, with studies confirming their ability to safely and effectively remove stratum corneum without damaging living cells (Park et al., 2023). This property has led to their widespread use in the development of personal care products targeting dandruff control, acne treatment, and skin brightening (Navone and Speight, 2018). Keratinases themselves are core components in gentle depilatory formulations, enabling the development of low-irritation hair removal and skincare products (Sanghvi et al., 2016). Furthermore, short peptides derived from keratin hydrolysis display antioxidant activity, making them suitable as natural antioxidant ingredients in cosmetic formulations (Zhang et al., 2023). Given their high efficiency in degrading insoluble keratin, novel M36 family keratinases identified using bioinformatics tools represent potential enzymatic resources for developing next-generation functional skincare products (Yan et al., 2024). Regarding technological integration, keratinase from *Pseudomonas aeruginosa*-C1M facilitates the guided synthesis and functionalization of antibacterial silver nanoparticles (AgNPs) (Saba et al., 2025). The resulting keratinase–nanocomplexes exhibit enhanced stability alongside combined antibacterial and antioxidant properties, thus significantly broadening the scope for developing multifunctional cosmetics (Sharma et al., 2023).

### 7.3 Incorporation of keratinases in detergents

Keratinases exhibit remarkable stability and can maintain their activity in the presence of surfactants, detergents, and various organic solvents (Ramakrishna Reddy et al., 2017). This characteristic underpins their significant market potential for application and development within the detergent industry. Several researchers have experimentally demonstrated that keratinases combined with detergents display high efficiency in stain removal, particularly in the removal of blood stains on clothing or cotton fabrics (Spyros, 2003; Zhang et al., 2016). Bokveld et al. identified a strain of *Chryseobacterium*, designated FANN1, that produced a keratinase retaining high activity even after co-incubation with various chemical reagents and commercial detergents. Compared to other proteases, this keratinase more effectively facilitates the removal of both soluble and insoluble protein-based stains from fabrics (Bokveld et al., 2021). Similarly, Nnolim et al. (2020) isolated *Arthrobacter* sp. KFS-1 from a landfill soil sample, a strain that also shows promising potential for detergent applications. Protein engineering strategies, such as rational design (e.g., introducing the N122Y point mutation) and directed evolution, can significantly enhance the thermostability and catalytic efficiency of keratinases, thus laying the groundwork for their use in high-temperature washing cycles (Liu et al., 2013). Immobilization techniques, such as the preparation of magnetic cross-linked enzyme aggregates (CLEAs), can further improve their operational stability and reusability, making them highly suitable for developing high-performance, low-cost industrial biocatalytic detergents (Lotfi et al., 2022). To date, researchers have identified keratinases with specialized tolerance profiles in diverse microorganisms, including a keratinase from *Purpureocillium lilacinum* that displays excellent tolerance to oxidizing agents (bleaches) (Cavello et al., 2012), serine keratinases possessing both thermostability and effective cleaning power (Akram et al., 2020), and a cold-adapted keratinase from *P. oxalicum* whose activity is enhanced in the presence of surfactants (Abdel-Latif et al., 2025). These high-performance keratinases, obtained through genetic engineering or natural screening, demonstrate exceptional robustness in complex washing environments, highlighting their broad prospects as next-generation biocatalysts for detergent formulations (Li et al., 2025).

## 8 Current challenges in the industrialization of keratinases

### 8.1 Low heterologous expression efficiency

Keratinases are often produced at low levels in their native hosts, and some wild-type strains may be pathogenic, rendering them unsuitable for industrial-scale production (Zhang et al., 2022). As noted by Yahaya et al. (2021), “recombinant keratinases are expressed as inclusion bodies, resulting in loss of keratinolytic function and complicating downstream processing”. Moreover, factors such as codon bias, weak promoter activity, and inefficient signal peptides further limit the high-level expression of keratinases (Akram et al., 2020). These expression challenges not only increase production costs but also lead to insufficient yields of active enzyme to meet industrial requirements, representing a major technical bottleneck in the large-scale application of keratinases.

### 8.2 Poor stability under industrial conditions

Keratinases in practical industrial settings are often required to withstand harsh conditions, including high temperatures, extreme pH levels, surfactants, metal ions, and organic solvents. However, the physicochemical properties of most natural keratinases fall short of industrial demands, constituting a major bottleneck for their widespread application. Currently reported natural keratinases typically exhibit optimal pH between 6.0 and 9.0, with >80% activity retained only within a narrow window of pH 7.0–8.5. Beyond this range, activity declines rapidly. Thermally, their optimum lies between 40 °C and 60 °C, yet their half-lives under sustained industrial temperatures are short—often below 2 h at 50 °C and under 30 min at 60 °C. Specific processes impose even stricter requirements: feed pelleting involves steam at 85–95 °C; leather dehairing operates at pH 10.0–11.5; and detergent applications combine temperatures of 50–60 °C with pH 9.5–11.0. These conditions demand exceptional thermostability and alkali tolerance (Navone and Speight, 2018). Furthermore, keratinases generally display poor tolerance to organic solvents, which limits their application in reaction systems involving organic phases. The inherent structural flexibility of the enzyme molecule and the susceptibility of its active site to disruption are fundamental causes of this instability, highlighting an urgent need for improvement through protein engineering strategies.

### 8.3 Insufficient substrate degradation efficiency and specificity

Native keratin, which is characterized by highly cross-linked disulfide bonds and compact structure, represents a recalcitrant substrate (Qiu et al., 2022). Most keratinases exhibit limited efficiency in degrading native keratinous materials (e.g., feathers and hair), often necessitating pretreatment or the addition of reducing agents to disrupt disulfide bonds and enhance degradation (Yi et al., 2020). It is noteworthy that reducing agents such as dithiothreitol (DTT) are frequently necessary during the degradation process; however, these agents may destabilize the enzyme’s native structure, leading to autolysis and the loss of enzyme activity (Sharma et al., 2023). Furthermore, keratinases from different sources show marked variations in their specificity toward *α*-keratin and *β*-keratin. Most enzymes degrade β-keratin (e.g., feathers) more effectively than α-keratin (e.g., hair and wool). This substrate specificity constrains their application in processing mixed keratinous waste. Developing keratinases with broad substrate adaptability or constructing multi-enzyme synergistic degradation systems represent promising strategies to address this challenge.

### 8.4 High production costs and immature process technology

Currently, the extended fermentation duration and low enzyme yield of keratinase production contribute to its high costs, hindering large-scale industrial adoption (Zhang et al., 2022). Current keratinase manufacturing primarily relies on laboratory-scale systems, with a lack of optimized fermentation processes tailored for industrial operation. Key parameters—such as carbon-to-nitrogen ratio, dissolved oxygen control, and induction strategies—have yet to be standardized. Moreover, crude enzyme preparations often contain additional proteases or impurities, which can compromise stability and efficacy in specific applications, necessitating the further optimization of purification and formulation protocols (Pei et al., 2025). Downstream processing is also challenged by low enzyme recovery rates and underdeveloped formulation technologies, which collectively drive up production expenses. From an economic standpoint, developing low-cost culture media, optimizing fermentation conditions, and establishing efficient downstream processing methods are crucial to reduce keratinase production costs.

### 8.5 Lack of efficient protein engineering and screening platforms

Although protein engineering techniques such as directed evolution and rational design show potential for improving enzyme performance, there is currently a lack of high-throughput, low-cost screening methods specifically adapted for keratinases. Enzyme engineering approaches based on non-canonical amino acids are limited in widespread application due to their high cost and technical complexity (Sharma et al., 2023). In addition, the diversity of keratinase activity assays and the absence of standardized protocols pose challenges for comparing results across different studies (Zhang et al., 2025). Existing screening methods, which predominantly rely on plate hydrolysis zones or microplate colorimetric reactions, suffer from low throughput and insufficient sensitivity, rendering them inadequate for the evaluation of large mutant libraries. Developing automated, miniaturized high-throughput screening platforms and establishing rapid, accurate activity assays are essential prerequisites for accelerating keratinase engineering.

### 8.6 Scarcity of commercial varieties and underdeveloped application ecosystem

To date, very few keratinase products have achieved commercialization, with the enzyme derived from *B. licheniformis* PWD-1 being one of the most widely used examples (Pei et al., 2025). Although a considerable number of patents related to keratinases have been filed, not all remain active, and many display overlapping research trends with limited innovation (Akram et al., 2020). The keratinase application ecosystem is not yet fully established, and disconnects persist within the industrial chain spanning from enzyme production to end-use applications. In potential industrial sectors such as feed, leather, and detergents, keratinases have yet to demonstrate clear advantages over conventional chemical treatments in terms of cost and efficiency. Furthermore, the insufficient development of specialized enzyme formulations tailored to specific application scenarios hinders their market penetration.

## 9 Discussion

In recent years, the application scope of keratinases has expanded from traditional fields, such as feed and leather processing, to emerging sectors, including biomedicine and bioenergy. This shift has been driven by the deeper integration of insights into their catalytic mechanisms and advances in technological development. The research paradigm in this field has undergone a revolutionary transformation, with AI-assisted design and synthetic biology tools emerging as core drivers for breakthroughs in keratinase performance. However, researchers remain clearly aware that cost and stability represent the primary obstacles to achieving the widespread commercialization of keratinases. Consequently, future studies will increasingly focus on interdisciplinary strategies to construct efficient, stable, and low-cost keratinase-based biomanufacturing platforms (Yan et al., 2025). The in-depth development and industrial application of keratinases should center on three key directions: protein engineering, AI-aided design, and establishing multi-enzyme synergistic catalytic systems. Integrating the latest research findings to achieve cohesive technological synergy will be essential to advance keratinase applications from laboratory research to industrial implementation.

### 9.1 In the field of protein engineering

Following the use of X-ray crystallography to elucidate enzyme structures such as MtaKer in *Thermus thermophilus* WR-220 and fervidolysin from *Fervidobacterium* species, targeted mutations at key regions, including calcium-binding sites and *β*-sandwich domains, have been shown to significantly enhance thermal stability and catalytic efficiency (Yan et al., 2025). Directed evolution techniques, exemplified by the error-prone PCR method, have yielded KerBp mutant T18, which exhibits 2.1-fold higher activity than the wild-type enzyme. Non-classical engineering strategies, such as fusion with the Propeptide C-terminal domain (PPC), enabled over 80% feather degradation within 8 h, offering an effective pathway for optimizing enzyme performance (Pei et al., 2025). A research team from Soochow University employed a three-step rational surface design approach to engineer a spore-displayed keratinase with substantially improved acid tolerance. The resulting variant efficiently converted feather meal in lactic acid bacteria-fermented feed and simulated gastric environments, thereby overcoming a key technical barrier in the development of feather-based feed (Das et al., 2024). Although significant progress has been made in existing research, the practical performance of keratinases in industrial application scenarios still requires further verification. For example, improvements in thermal stability achieved under laboratory conditions may face additional challenges in the complex environments of actual industrial production. Notably, keratinases obtained from different sources exhibit considerable variability in performance under industrial conditions, highlighting the need to establish a more comprehensive enzyme performance evaluation system.

### 9.2 In the field of AI-assisted design

In the field of artificial intelligence (AI)-assisted design, tools such as AlphaFold 2 and machine learning (ML) models are overcoming the limitations of conventional protein engineering and have become key drivers for optimizing keratinase performance. Traditional protein engineering faces several constraints: three-dimensional structure determination often relies on labor-intensive, low-throughput experimental methods; mutation screening is largely empirical and inefficient; and strain optimization lacks precise molecular guidance for targeted improvements. AlphaFold^2^ enables the accurate prediction of three-dimensional keratinase structures, and its integration with molecular dynamics simulations facilitates the identification of flexible regions associated with thermal stability. For example, mutation at the N122 site in a *B. licheniformis* keratinase resulted in a 5.6-fold increase in catalytic activity (Wang et al., 2023). Machine-learning models, trained on large keratinase datasets, can establish sequence–structure–function relationships and reliably predict mutation effects. This data-driven approach addresses the low efficiency of traditional screening and supports multi-objective optimization tailored to industrial needs. Furthermore, AI-driven mutation screening with CRISPR-Cas9 genome editing technology has tripled enzyme production in *B. licheniformis*. Multi-omics analysis has further elucidated the regulatory network of the Keratinase gene A (KerA) gene cluster, providing molecular insights for strain optimization (Yan et al., 2024).

### 9.3 Multi-enzyme synergistic catalytic systems

Multi-enzyme synergistic catalytic systems significantly enhance degradation efficiency through employing a functional division of labor among reductase–endopeptidase–exopeptidase to disrupt the recalcitrant structure of keratin. For example, the synergistic action of T3 *γ*-glutamyltranspeptidase and subfamily B (S8B) serine endopeptidase from *Bacillus* sp. CN2 achieved an 86.7% feather degradation rate (Yan et al., 2025). A microbial consortium containing *Lysinibacillus* and other strains demonstrated a 96% degradation rate for feathers (Yan et al., 2024). Combining keratinases with cellulases and lipases increased the resource utilization rate of mixed waste by 40% (Akram et al., 2022). Furthermore, the synergy between thermostable keratinases and reductases has been shown to enhance the degradation of feather-based pollutants, thereby expanding potential application scenarios (Bj and Dhanasingh, 2025).

### 9.4 Comparison of different engineering strategies and economic feasibility analysis

The selection of protein engineering strategies profoundly influences the performance, stability, and ultimately, the economic feasibility of keratinases in industrial settings. A rational choice among available approaches is therefore crucial to advance their commercialization. Table 4 summarizes the comparative advantages and disadvantages of the three principal engineering strategies: directed evolution, rational design, and AI.

**Table 4:** Comparison of protein engineering strategies for keratinase improvement.

Strategy	Advantages	Disadvantages
Directed evolution (e.g., error-prone PCR)	Structure-independent Low technical barrier Can yield unexpected beneficial mutations	Random, low-throughput, costly screening Uncontrolled mutagenesis Poor for multi-objective optimization
Rational Design	Highly targeted; low screening cost Addresses specific performance traits	Requires high-resolution structure High expertise needed; limited design scope May disrupt enzyme conformation
AI-Assisted design	Efficient and accurate mutation prediction Enables multi-objective and large-scale optimization	Relies on big data & high computing power Requires experimental validation High initial investment; less accessible to SMEs

The economic viability of keratinase commercialization hinges on a balance between production cost, operational stability, and application efficiency. Directed evolution, while powerful for trait discovery, incurs significant screening costs and is less suited for large-scale, multi-parameter optimization. Rational design offers precision but is bottlenecked by the need for solved structures and may have limited generality. In contrast, AI-assisted design, despite substantial upfront investment in data and computation, promises superior long-term cost-effectiveness. By enabling rapid, accurate prediction of optimal variants, it reduces iterative experimental cycles and can be scaled across multiple enzyme properties, amortizing initial costs over broader applications (Wang et al., 2023; Yan et al., 2024). Enzyme stability under industrial conditions (e.g., high temperature, extreme pH, surfactants) is a critical determinant of application cost. Instability leads to frequent re-dosing or enzyme replacement, increasing operational expenses. Protein engineering (including AI-assisted methods) and enzyme immobilization (e.g., CLEAs) are key to enhancing robustness, thereby lowering the effective cost per unit of processing (Liu et al., 2013; Lotfi et al., 2022). As demonstrated by Yan et al. (2024), strategic engineering to improve keratinase stability and catalytic efficiency directly reduces the cost burden in agricultural and industrial applications.

In conclusion, no single strategy is universally optimal. Future development should adopt a synergistic, scenario-specific approach: using AI and rational design to guide intelligent library construction, followed by directed evolution for experimental validation. Combining these strategies with immobilization and process optimization will be essential to achieve the dual goals of high performance and economic feasibility, ultimately accelerating the industrial adoption of keratinases for sustainable waste valorization and green manufacturing.

The deep integration of these three core strategies, when combined with immobilization technologies such as graphene oxide-based supports, which enable enzyme reuse for over 15 cycles, and green process optimization (e.g., enzymatic dehairing, which reduces energy consumption by 50–70% compared to chemical methods), can promote the large-scale application of keratinases in environmental management (e.g., biogas production from waste feathers) and sustainable agriculture (e.g., the production of highly digestible feather meal feed). This integration provides core technological support for achieving the goals of a circular bioeconomy (Yan et al., 2025; Bj and Dhanasingh, 2025). Although keratinases have not yet been widely adopted in industrial production, their demonstrated technological potential and application prospects can no longer be overlooked. From the precise regulation of enzyme performance via protein engineering to the efficiency leap enabled by AI-aided design, and further to the systematic solutions offered by multi-enzyme synergistic catalysis, these research breakthroughs not only pave the way for the industrialization of keratinases but also reveal a transformative trend in biomanufacturing, shifting from single-enzyme optimization to innovation across the entire technological chain. In the future, with the deeper integration of interdisciplinary technologies and the accumulation of industrial-scale experience, researchers are expected to achieve key breakthroughs in reducing keratinase production costs and enhancing their operational stability. Ultimately, this will facilitate the transition from laboratory research to industrial application, providing essential technological support for resource recycling and sustainable development.

### 9.5 Limitations

While this review utilized a defined search strategy, some limitations should be acknowledged. Firstly, as a narrative review, the literature selection process, though described, was not conducted as a fully systematic review with multiple independent screeners. This may introduce selection bias, where the synthesis and emphasis of findings could be influenced by the authors’ perspective. Secondly, studies on microbial keratinase sources often focus on strains isolated from specific ecosystems or geographic regions (e.g., poultry farms, specific thermal springs). This geographic and ecological bias in the primary literature may limit the generalizability of the summarized enzymatic properties and optimal conditions, as keratinases from underrepresented environments might possess distinct characteristics.

## 10 Conclusion

This review comprehensively examines the current state of keratinase research, encompassing keratinase sources, mechanisms of action, enzymatic properties, and applications across various industries. Keratinases represent a class of hydrolytic enzymes capable of specifically degrading recalcitrant keratin. Their applications can be broadly divided into two categories. First, due to their exceptional substrate adaptability, keratinases demonstrate significant biotechnological potential in the conversion of keratinous waste such as feathers and hair into high-value products. This enables the effective degradation of keratin-rich waste, achieving the dual objectives of environmental protection and resource recycling. Examples include the use of keratinases for dehairing (Akhter et al., 2020) and transforming keratinous waste into biogas (Yamamura et al., 2002), providing innovative solutions for a green circular economy (Singh et al., 2016). Second, extending beyond environmental management, keratinases are incorporated into industrial products to enhance their efficacy. In the feed industry, keratinases are applied to pretreat animal feed, thus improving protein digestibility and nutritional value, thereby supporting the development of unconventional feed resources (Xu et al., 2022). In the cosmetics sector, keratinase hydrolysates, including bioactive peptides and amino acids, serve as functional ingredients in skincare products, highlighting their potential in manufacturing high-value-added goods (Brandelli et al., 2015). In conclusion, keratinase technology is emerging as a pivotal link in advancing the circular bioeconomy, enabling the conversion of waste protein resources into energy, materials, and chemicals, thereby offering a viable biocatalytic pathway toward achieving a sustainable “waste-to-resource-to-product” closed loop.

### 10.1 Prospects

The primary challenge in this area lies in the mass production of keratinases with high degradation efficiency. Two main strategies are currently being implemented to address this issue. The first involves employing molecular approaches targeting promoters, chromosomal integration, and signal peptides to enhance keratinase yields. For example, Gong et al. systematically evaluated 10 different promoters in *B. subtilis* to improve protease production. Under identical experimental conditions, the strain carrying the strong alkaline protease gene (*aprE*) promoter achieved approximately 15-fold higher enzyme yield compared to the control (Gong et al., 2020). However, this production level still fails to meet large-scale market demands. The second strategy focuses on the heterologous expression of cloned keratinase genes. This methodology involves screening high-activity keratinase-producing strains, cloning the corresponding genes, conducting heterologous expression, and subsequently characterizing the optimal temperature, pH, and the influence of metal ions (Wang et al., 2019; Fu, 2021). *Escherichia coli*, *Bacillus* species, and *Pichia pastoris* are frequently employed as heterologous expression hosts due to their demonstrated efficacy (Yahaya et al., 2021). Nevertheless, several challenges persist in the heterologous expression of keratinases, including yield dependency on expression conditions, potential host pathogenicity (Jaouadi et al., 2010), the formation of inclusion bodies (Ben Elhoul et al., 2016), and potential declines in enzymatic activity post-expression, all of which significantly impede commercial development.

The secondary challenge concerns maintaining keratinase activity under complex application conditions. Today, two primary approaches are being pursued to address this limitation. First, superior keratinases can be created through chemical modification and molecular engineering (Gao, 2017). In industrial production, immobilization treatment is commonly employed to enhance the stability and efficiency of enzymes (Latip et al., 2021). For example, Fang et al. (2016) identified a unique PPC in the keratinase *Stenotrophomonas maltophilia* BBE11-1 (KerSMD) and demonstrated that the truncation of this C-terminal sequence enhanced catalytic efficiency, thermophilicity, salt tolerance, and detergent compatibility. Second, the optimization of various cultivation parameters enables screening for improved enzyme properties. In previous work, Akram et al. (2020) isolated a novel bacterial keratinase from *B. subtilis* NKSP-7 that exhibited remarkable stability and compatibility with commercial detergents, metal ions, organic solvents, reducing agents, and surfactants. Additionally, functional screening coupled with gene sequencing technologies has facilitated the identification of novel keratinases with enhanced activity profiles (Su et al., 2020). Given the cross-disciplinary applications of keratinases, fundamental and applied research on keratinases has become a focal point in industrial biotechnology. Research directions such as rational protein design, efficient heterologous expression, and immobilization process development align closely with sustainable development requirements, displaying continued and profound research significance (Bornscheuer et al., 2012; Adrio and Demain, 2014).

## Glossary

AI: Artificial intelligence
CNKI: China National Knowledge Infrastructure
PH: Potential of hydrogen
PMSF: Phenylmethylsulfonyl fluoride
EDTA: Ethylenediaminetetraacetic acid
PCR: Polymerase chain reaction
CUPP: Conserved unique peptide pattern -based enzyme family classification tool
TSS: Total suspended solids
TDS: Total dissolved solids
COD: Chemical oxygen demand
SEM: Scanning electron microscopy
DCCA: Dichloroisocyanuric acid
BOD: Biochemical oxygen demand
M36: Metalloprotease family 36
AgNPs: Silver nanoparticles
CLEAs: Cross-linked enzyme aggregates
DTT: Dithiothreitol
PPC: Propeptide C-terminal domain
KerA: Keratinase gene A
S8B: Subfamily B
aprE: Alkaline protease gene
KerSMD: keratinase *Stenotrophomonas maltophilia* BBE11-1
